# Kenya National Hospital Insurance Fund Reforms: Implications and Lessons for Universal Health Coverage

**DOI:** 10.1080/23288604.2018.1513267

**Published:** 2018-11-06

**Authors:** Edwine Barasa, Khama Rogo, Njeri Mwaura, Jane Chuma

**Affiliations:** 1 Health Economics Research Unit, KEMRI–Wellcome Trust Research Programme, Nairobi, Kenya; 2 Centre for Tropical Medicine, Nuffield Department of Clinical Medicine, University of Oxford, Oxford, UK; 3 The World Bank Group, Kenya Country Office, Nairobi, Kenya

**Keywords:** efficiency, equity, social health insurance, universal health coverage

## Abstract

This article identifies and describes the reforms undertaken by the National Hospital Insurance Fund (NHIF) and examines their implications for Kenya’s quest to achieve universal health coverage (UHC). We undertook a review of published and grey literature to identify key reforms that had been implemented by the NHIF since 2010. We examined the reforms undertaken by the NHIF using a health financing evaluation framework that considers the feasibility, equity, efficiency, and sustainability of health financing mechanisms. We found the following NHIF reforms: (1) the introduction of the Civil Servants Scheme (CSS), (2) the introduction of a stepwise quality improvement system, (3) the health insurance subsidy for the poor (HISP), (4) revision of monthly contribution rates and expansion of the benefit package, and (5) the upward revision of provider reimbursement rates. Though there are improvements in several areas, these reforms raise equity, efficiency, feasibility, and sustainability concerns. The article concludes that though NHIF reforms in Kenya are well intentioned and there has been improvement in several areas, design attributes could compromise the extent to which they achieve their intended goal of providing universal financing risk protection to the Kenyan population.

## Background

Low- and middle-income countries (LMICs) are increasingly adopting universal health coverage (UHC) as their health policy priority.^1^ To achieve UHC, countries must expand the range of services they provide to their citizens, expand population coverage with a prepayment mechanism, and reduce the proportion of direct costs that citizens pay to access health care services.^2^ Kenya has made a commitment to achieve UHC by 2022. The country has a mixed health financing system that is financed by revenues collected by the government (national and county) through taxes and donor funding, the National Hospital Insurance Fund (NHIF) through member contributions, private health insurance companies through member contributions, and out-of-pocket spending by citizens at points of care.^3^ Purchasing of health care services is carried out through (1) supply-side subsidies to public facilities by national and county governments—for instance, the county departments of health provide line budgets to county hospitals to finance service delivery to citizens within the county; (2) the NHIF, which contracts public and private health care facilities in Kenya and pays them for services provided to its enrolled members; and (3) private health insurance companies that contract private health care facilities and pay them for services provided to their enrolled members.^3^ Table 1 outlines the country’s key health financing indicators.

Contributory health insurance has gained popularity as a health financing mechanism in Kenya and other LMICs, reforming their health systems for UHC.^4^ An increasing number of sub-Saharan African countries have either established or are in the process of establishing a contributory public health insurance scheme. For example, Ghana, Kenya, Nigeria, Rwanda, and Tanzania have contributory public health insurance schemes, and South Africa, Swaziland, Lesotho, Sierra Leone, Liberia, Zambia, Uganda, Bukina Faso, and Zimbabwe are considering establishing one.^4–6^ The Kenyan government has made a decision to use the NHIF as one of the key strategies for scaling up population coverage with a prepayment health financing mechanism.^7^

The NHIF is a public institution that was established in 1966 to provide mandatory health insurance to formal sector employees, and its mandate later expanded to cover informal sector workers in 1998.^8^ Membership in to the NHIF is mandatory for formal sector workers, who pay an income rated monthly contribution through statutory deductions, whereas it is voluntary for informal sector workers, who pay a flat rate contribution directly to the NHIF. Previous analysis has shown that NHIF’s purchasing is passive rather than strategic.^7^ Health insurance coverage in Kenya is generally low (19%; Table 1). The NHIF is the main health insurer in Kenya, covering 16% of Kenyans, whereas the 32 private health insurers collectively cover a mere 1% of the Kenyan population.^9^

In efforts to enhance the NHIF’s capacity to deliver the promise of UHC to Kenyans, the Kenyan government has introduced several reforms in the last eight years. In this article, we analyze the implications of these reforms for Kenya’s quest to achieve UHC. We focus on the entire range of recent reforms given that they are linked and aimed at the same objective: increasing population coverage with the NHIF to increase access to quality health care services while offering protection from the adverse effects of out-of-pocket payments. This analysis adds to the literature on health financing reforms in LMICs by illuminating Kenya’s experience with implementing health insurance reforms and providing lessons on how the configuration of such reforms can influence progress toward UHC. This experience and lessons are relevant not only for Kenya but for other LMIC settings that either have or are planning to introduce a contributory health insurance mechanism.

## Methods

### Study Approach

We reviewed both peer-reviewed publications and grey literature that contained information on the NHIF. To obtain peer-reviewed and grey literature, we conducted Google searches and a search in Google Scholar and PUBMED using the following keywords: “national hospital insurance fund Kenya,” “NHIF Kenya,” “NHIF reforms Kenya,” and “NHIF policies Kenya.” We also specifically searched on the websites of the Kenyan Ministry of Health, NHIF, key international development organizations that support the Kenyan Ministry of Health and NHIF on health financing initiatives (the World Bank Group, German Development Corporation, United States Agency for International Development, and World Health Organization) and an online database of Kenyan laws. We restricted our search to documents and papers that were published from 2010 onwards. We chose 2010 as our stating point because this was the year that the report of a strategic review of the NHIF commissioned by the Kenyan Ministry of Health and the International Finance Corporation (IFC); hereafter refered to as the strategic review was released. The strategic review assessed the performance of the NHIF in the period preceeding 2010. Further, the period after 2010 is the period during which substantial reforms were implemented by the NHIF. We only included documents, reports, and peer-reviewed papers that contained information relating to NHIF reforms and/or a description of NHIF operations or performance. We identified seven peer-reviewed papers and 16 grey literature. Table 2 outlines the documents identified and reviewed.

### Analytical Framework

To analyze the information obtained from the retrieved documents, we applied the framework proposed by McIntyre^30^ for assessing health financing mechanisms. This framework proposes that health financing mechanisms should be assessed on their feasibility, equity, efficiency, and sustainability.^30^ The McIntyre framework offers a range of feasibility considerations for health financing mechanisms. These include actor/political support or opposition to aspects of revenue collection, risk pooling, and purchasing; the feasibility of collecting funds (willingness and/or ability of citizens to make contributions); and whether there is adequate capacity (such as technical, administrative, resources) to ensure successful implementation.^30^ With regards to equity, there is general agreement that individuals should contribute to health care according to their ability to pay and benefit according to their need for care.^31^ An equitable health financing system will therefore involve cross-subsidies from the rich to the poor and from the healthy to the ill.^30^ With regards to efficiency, revenue collection of a health financing mechanism is efficient if it generates a relatively large amount of funds while minimizing collection costs.^32^ Efficiency is assessed based on how aspects of revenue collection, pooling, and purchasing influence technical and allocative efficiencies. For instance, the resource allocation mechanism of a health financing mechanism is technically efficient if it provides resources to the maximum number of fundable services and is allocatively efficient if resources are allocated to services addressing the heaviest burden of ill health in the community for which effective interventions exist, while giving priority to the most cost-effective interventions.^30^ A health financing mechanism is considered sustainable if it has long-term stability and potential for generating revenue.^30^ Sustainable financing mechanisms should not be subject to considerable and frequent fluctuations.^30^

A limitation of our selected approach and framework is that it does not examine the effect of NHIF reforms against the ultimate UHC goals of effective service coverage and financial risk protection. This is due to a lack of data that could inform this analysis that are specific to NHIF reforms. However, the criteria outlined in the McIntyre framework reflect health financing configurations that are instrumental in attaining the ultimate UHC goals and hence an analysis using this framework is informative regarding whether the direction of health financing reforms is appropriate. Another limitation is the inability to causally attribute reforms to effects. We used this framework to carry out a qualitative assesment of the reforms rather than a quantitiative impact assessment of each of the reforms against the four framework criteria.

## Results

The strategic review commissioned to assess the performance of NHIF in relation to its existing mandate identified several weaknesses.^8^ Among others, the strategic review revealed that health insurance coverage by the NHIF in Kenya was low and that informal sector membership was characterized by high attrition rates.^8^ The report further highlighted that the NHIF was inefficient, with a benefit payout rate of 55% and a proportion of administrative costs of 45% in 2010.^8^ The report made recommendations for reforms in five key areas of the NHIF^8^: (1) policy and regulatory framework, (2) governance, (3) financial sustainability, (4) effectiveness, and (5) efficiency. Interested readers can refer to the report for further details on its findings and recommendations. Since then, the NHIF has embarked on several reforms. Given that NHIF membership is mandatory for individuals in the formal sector and voluntary for individuals in the informal sector, the strategic review recommended that efforts to scale up NHIF coverage should focus on enrolling informal sector individuals. This focus is reflected in the NHIF strategic plan^24^ and in the NHIF informal sector strategy.^26^ Most of the reforms implemented by the NHIF since 2010 (other than the introduction of the Civil Servants Scheme) are hence aimed at expanding membership coverage with a specific focus on the informal sector.^33^ In this section, we will begin by describing the key reforms undertaken by the NHIF since 2010, followed by an analysis of the implications of the reforms for UHC in Kenya.

### Reforms Undertaken by the NHIF

#### The Introduction of the Civil Servants Scheme

In 2012, the NHIF introduced an insurance scheme for formal sector government workers and their dependents (civil servants) known as the Civil Servants Scheme (CSS).^34,35^ Under the CSS, the Kenyan government remits the medical allowances, previously paid directly to civil servants, to the NHIF as premium contributions.^34^ Funds for the CSS are managed separately from other NHIF funds, and beneficiaries enjoy a wider benefit package,^34^ including comprehensive outpatient and inpatient services accessed through contracted health care providers. Since the inception of CSS, civil servants have successfully negotiated for expansion of the benefit package to include treatment abroad and land ambulance and airlifting services.^19^ Civil servants and their dependents are capitated to their preferred health care provider at a rate of 1,500 Kenya shillings (KES; 15 USD) per annum for public facilities, and 2,850 KES (28.5 USD) for private facilities.^34^ The different rates account for supply-side subsidies received by public facilities from the government through annual budgetary allocations. Approximately 600,000 civil servants and their dependents are registered under this scheme.

#### Introduction of a Stepwise Quality Improvement System

In 2013, the NHIF, with financial support from the IFC and technical support from the PharmAccess Foundation, introduced the SafeCare quality improvement system.^17^ SafeCare aims to support basic health care providers in resource-restricted settings to go through stepwise structured improvement programs to deliver safe and quality-secured care to their patients according to internationally recognized standards.^17^ This differs from traditional quality assurance mechanisms that have a dichotomous approach to quality standards and hence allows small, poorly resourced health care facilities to implement a quality improvement plan with the goal of meeting the required standards for accreditation and contracting by the NHIF to provide health care services.

#### The Health Insurance Subsidy for the Poor

Another strategy adopted to expand population coverage with the NHIF and improve equity in coverage was the introduction of a health insurance subsidy for the poor (HISP) program.^16^ In April 2014, the Kenyan government launched the HISP pilot program—a comprehensive, fully subsidized, health insurance program for selected poor orphans and vulnerable children—benefiting from the government’s cash transfer program.^16,36^ The HISP pilot targeted 23,000 households (approximately 142,000 individuals) across the country for two years, with plans to progressively scale up coverage to the poorest 10% of the population.^16,36^ These households were selected from the poverty list of orphans and vulnerable children developed and maintained by the country’s Ministry of Labor, Social Security, and Services.^16,22^ Those on the list were targeted using a combination of proxy means and community verification.^16,22^ In August 2016, the HISP program was scaled up to approximately 170,000 households (approximately 600,000 individuals). HISP beneficiaries receive comprehensive services from contracted public and private providers.^36^ At the time of its launch, the NHIF did not cover outpatient services, with the exception of the CSS. However, to provide adequate financial risk protection, an outpatient package was specifically designed for the HISP beneficiaries. Although the HISP benefit package was much narrower than that of the civil servants,^36^ the capitation rate payable to contracted providers remained the same.

#### Revision of Monthly Contribution Rates and Expansion of the Benefit Package

In April 2015, the NHIF increased contribution rates for its national scheme members (Table 3), to account for increased cost of service provision and to expand the benefit package.^37^

Prior to this revision, the NHIF premiums were last revised in 1988.^11^ The monthly contributions for the lowest paid formal employee increased by 400%, and rates for the highest earners increased by 431%. Contribution rates for the informal sector increased by 213%.^37^ This increase was accompanied by expansion of the benefit package to include outpatient services and a range of what the NHIF labels special packages that include chronic diseases, surgical care, chemotherapy, renal dialysis, kidney transplant, and magnetic resonance imaging and computed tomography scans.^19,38^

Compared to the CSS, contracted public providers receive a lower annual capitation rate of 1,200 KES for public providers and 1,400 KES for private providers.^28^ Additionally, facilities are reimbursed separately for the special packages as outlined in Table 4.

#### The Upward Revision of Provider Reimbursement Rates (2016)

In March 2016, the NHIF increased the inpatient reimbursement rates following negotiations with health providers, as a means to reduce the proportion of direct costs payable by its members for inpatient care.^19,39^ For example, reimbursement for a normal delivery increased from 6,000 KES to 10,000 KES, and the daily rebate for inpatient care in a public facility doubled, from 600 KES to 1,200 KES.^39^ Though health providers expressed their dissatisfaction with the lower capitation rates, they agreed to provide outpatient services if the NHIF increased inpatient and special package reimbursement rates (Table 3).

### Implications of the NHIF Reforms for UHC

#### Feasibility

**Figure 1:**
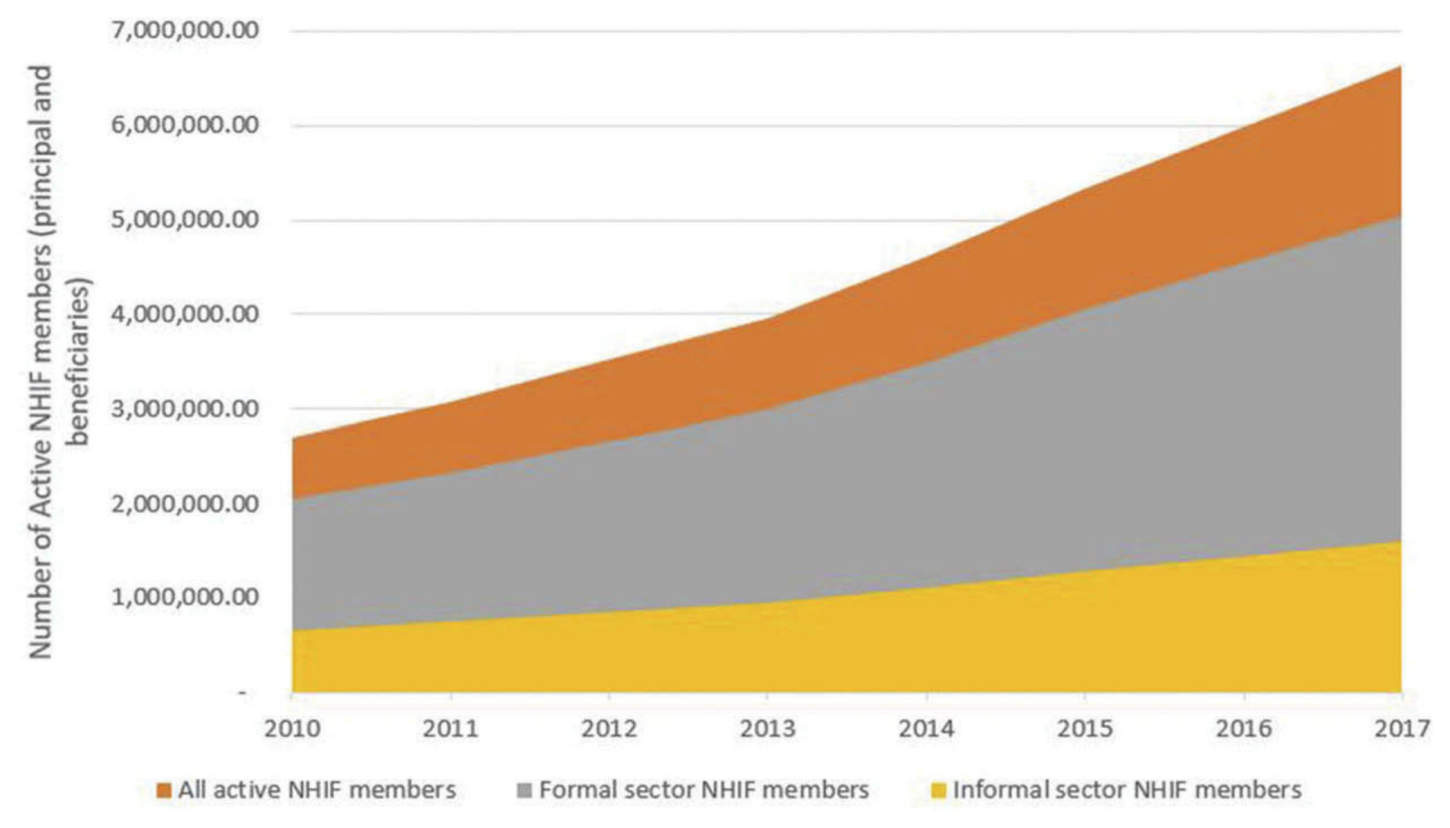
Absolute Number of Kenyans Enrolled in the NHIF

The first feasibility concern is the push to expand coverage using a voluntary contributory mechanism. In absolute numbers, the number of Kenyans (principal members plus beneficiaries) enrolled in the NHIF increased from about 2.7 million in 2010 to 6.6 million in 2017, the number of NHIF members who belong to the formal employment sector increased from 2 million to 2.5 million, and the number of informal sector employees increased from 652,000 to 1.6 million between 2010 and 2017 (Figure 1).^23,25,27,28^

**Figure 2:**
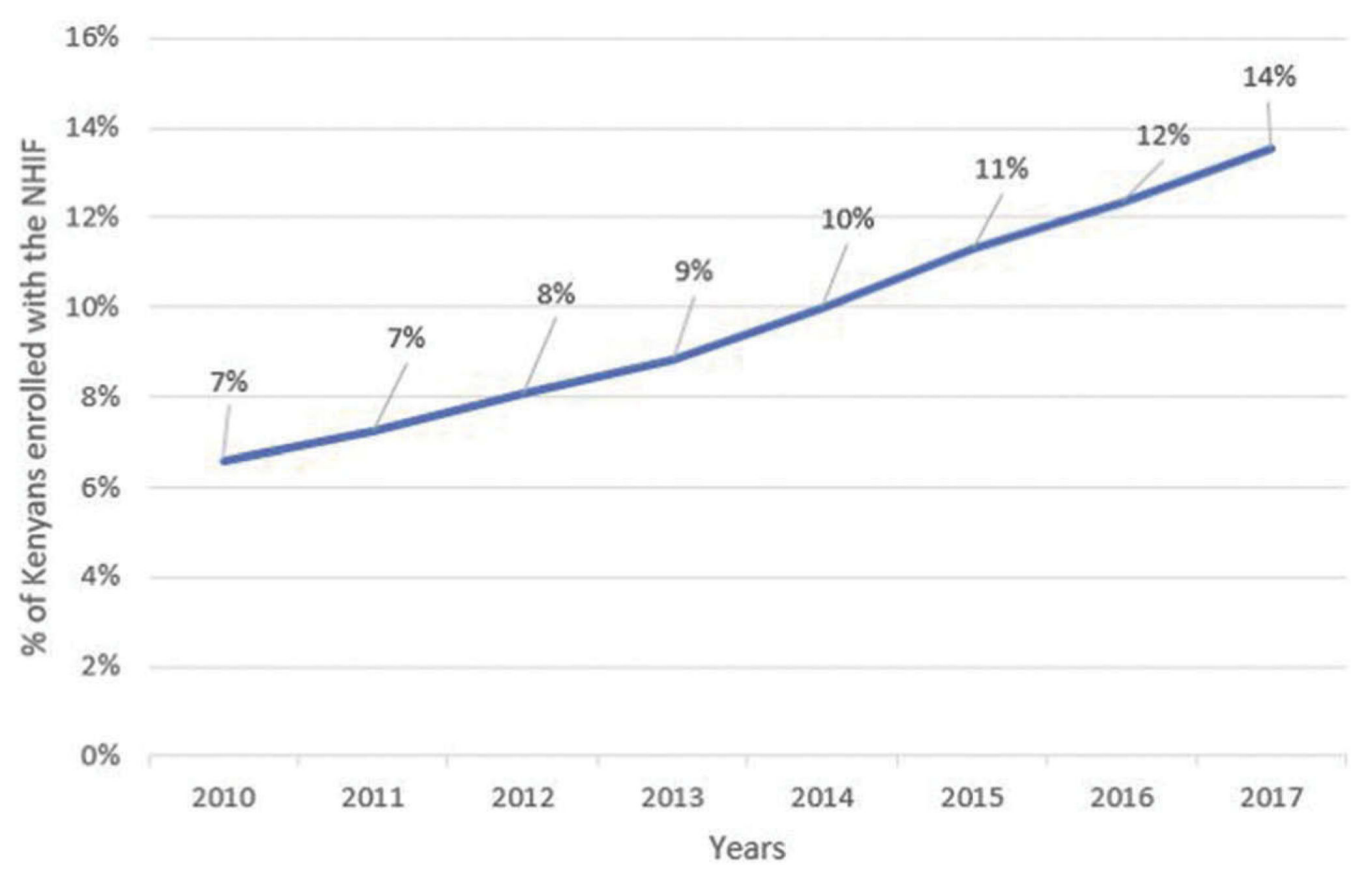
Changes in Population Coverage by the NHIF in Kenya

Computations from NHIF administrative reports show that despite an increase in the proportion of the Kenyan population enrolled in the NHIF between 2010 and 2017, the level of health insurance coverage by the NHIF remains low (Figure 2).^23,25,27,28^ These numbers computed from NHIF administrative data are in the same range as estimates computed from nationally representative household surveys.^9,40^

International experiences show that few countries have made substantial progress toward UHC on a voluntary basis.^4,41^ Kenya, like most LMICs, has a large proportion of informal sector workers. The challenge with scaling up voluntary health insurance among the informal sector is already evident. It is not surprising that at 19%, health insurance coverage in Kenya closely mirrors the proportion of formal sector workers. Though informal sector individuals form 83% of total employed individuals in Kenya,^42^ they contributed only 24% of the total number of individuals enrolled in the NHIF in 2017.^28^ Further, in 2017 the proportion of enrolled informal sector individuals who subsequently did not renew their membership was 73%,^28^ signaling a high attrition rate. Enrollment and retention among the informal sector using a voluntary contributory mechanism is problematic for several reasons.^4,41^ One, a significant proportion of informal workers are less well off compared to formal sector workers and therefore have a lower ability to pay for health insurance.^43,44^ Second, given that the informal sector is not organized in sizeable groups, it is administratively difficult to recruit, register, and collect regular contributions in a cost-effective way. Membership and premium payments are therefore often voluntary, leading to low uptake and poor retention.^4,45^ Third, informal sector worker incomes are often unpredictable,^4^ which makes it difficult to collect premiums regularly and increases attrition rates among this population.

The second feasibility concern relates to service provision constraints. Creating an entitlement to service benefits (whether comprehensive or limited) does not guarantee access to these services,^41^ unless a strong and well-distributed service delivery system is in place.^41^ The capacity of the purchasing organization and whether or not it engages in strategic purchasing is also critical.^41^ It has been reported that though the de jure NHIF benefit package was comprehensive, the range of benefits that its members de facto received was limited because certain services were often not available from the health care providers that NHIF had contracted to provide services to its members.^11,19,35,46^ This included medicines, laboratory, and radiological tests. Although the NHIF has clearly embarked on an ambitious plan to expand both the breadth and depth of coverage, this needs to be matched by increased capacity to support such reforms. A weak link in the NHIF system is the number and type of providers contracted and the quality of services provided to its members.^11,19,35,14^ Though the NHIF expanded its contracted health care facility network from 675 in 2010 to 4,011 in 2018, this is still only 40% of the total number of health care facilities in Kenya.^19^ One of the barriers to expansion of the NHIF health facility network is the slow and cumbersome health care facility empanelment process.^16,14^ For example, access to health care services by HISP beneficiaries was compromised by, among others, the slow empaneling and contracting of health care facilities.^16^ The NHIF also has weak capacity to monitor and enforce contracts, including mechanisms to assess quality of services offered to their members.^19,35^

The third feasibility concern is the implementation challenges and scalability of the HISP program. These include (1) the capacity to carry out poverty targeting to identify beneficiaries of the HISP program (the poverty list developed and maintained by the Ministry of Labor has only about 600,000 poor individuals, whereas the estimated number of poor Kenyans is 17 million^16^); (2) weak communication and hence low awareness among beneficiaries of their entitlement and how to access services; and (3) slow contracting of health care facilities by the NHIF.^16^ These challenges perhaps contributed to the finding of the HISP impact evaluation that there was no statistically significant effect of the HISP program to either health care utilization or level of out-of-pocket payments by HISP beneficiaries.^47^

#### Equity

**Figure 3:**
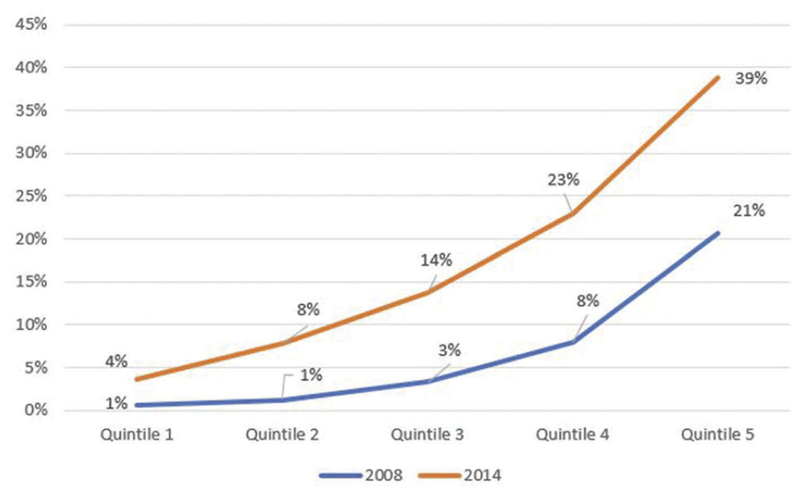
Trends in Health Insurance Coverage in Kenya by Socioeconomic Quintile^9^

The expansion of services among the informal sector and the introduction of the HISP program would ideally improve equity. However, several key design features of NHIF reforms raise equity concerns. First, the decision to first expand coverage to civil servants, who represent a sizeable number of the well-off population, undermines fairness and equity. This inequity is especially so because a sizeable proportion of Kenya’s population is in the informal sector, and 36% of the population lives below the national poverty line.^48^ As expected, health insurance coverage is skewed in favor of the rich (Figure 3).^9^ An analysis of benefits paid by the NHIF reveals that the per capita (per enrolled individual) benefits paid by the NHIF for members of the civil servants scheme is six times (60 USD) more than that paid for members of the national scheme (11 USD).

Second, feasibility challenges discussed previously have compromised the intention to enhance equity through the introduction of the HISP program. An analysis of the baseline data of the HISP beneficiaries revealed high levels of inclusion errors. This analysis reported that 65% of HISP beneficiaries were in the richest two quintiles (quintiles four and five) when their asset index is mapped onto the asset index scores in a nationally representative household survey data set (Kenya National Demographic and Health Survey).^18^

Third, expansion of the benefit package for both the CSS and the national scheme increases service coverage, albeit with equity implications. Given that it is unlikely that the NHIF will significantly increase membership among the informal sector and the poor, the increase in the benefit package will only benefit formal sector workers. By increasing benefits, the NHIF is implicitly trading off population coverage for greater benefits. This is because there is an expansion of services without an expansion of population coverage and yet the current covered population is predominantly composed of the well-off. Moreover, because the burden of disease is likely to be higher among the poor population, this trade-off further exacerbates inequities in access to health services. There is evidence that the different benefit packages for civil servants and the rest of the population not only create perceptions of unfairness among NHIF members^11^ but incentivize health care facilities to preferentially treat civil servants and discriminate against the rest of the population.^19,20^ For instance, it has been shown that one of the reasons informal sector individuals did not want to enroll in the NHIF was because they felt that the NHIF prioritized civil servants.^11^ It has also been shown that health care facilities preferentially allocated resources to civil servants by setting up, staffing, and equipping special civil servant clinics in hospitals at the expense of the rest of the service areas that served non–civil servants^20^ and preferentially treated civil servants by, for instance, letting them jump queues at the expense of non–civil servants.^19^

International experiences show that expanding coverage to the well-off and the formal sector first exacerbates inequalities and impedes countries’ progress toward UHC.^4,41^ Indeed, one of the unacceptable trade-offs highlighted by the World Health Organization’s Consultative Group on Equity and UHC relates to providing universal coverage to those with the ability to pay, while excluding informal workers and the poor.^49^ As Kenya makes the difficult choices related to what services to provide and to whom and the extent of financial risk protection, it is important that such decisions ensure fairness and equity. Providing varying benefit packages, not by virtue of need but by ability to pay, may not only promote inequities in access to health services but potentially promote inequities in health outcomes.

Fourth, voluntary health insurance is regressive. An earlier analysis of the NHIF premiums showed that contributions for both formal and informal sector workers were regressive.^50^ The revised premiums, though well intended, increased the regressivity of contributions among both formal sector individuals by broadening the income bands and informal sector individuals by increasing the contribution rate of a flat rate premium. It is important that any efforts to revise the design of NHIF premiums ensure that progressivity is maintained.

Fifth, the upward revision of the NHIF premium contribution rates is unaffordable to informal sector individuals. Informal sector individuals have expressed concern about the affordability of the revised premium contribution rate (500 KES per month).^11,13,19^ Further, a study on willingness and ability to pay the NHIF premium by the informal sector showed that the new rate was unaffordable for 75% of this population group.^15^

Sixth, contracting of health care facilities to provide services to NHIF members is biased in favor of urban facilities, predominantly hospitals, rather than small outpatient facilities that provide primary health care.^19^ The poor typically reside in rural regions and tend to use smaller outpatient facilities, rather than hospitals and/or facilities in urban areas. This bias therefore promotes inequities in access to services.

Seventh, the NHIF signs different contracts with the same health care facilities depending on the scheme and benefit package. These contracts have overlapping provider payment mechanisms but different payment rates and service entitlements. For example, whereas the NHIF pays an annual capitation rate of 2,850 KES for members of its CSS, it pays the same facility an annual capitation rate of 1,200 KES for outpatient care for the general population. Similarly, whereas the NHIF reimburses the full cost of delivery for the civil servants based on a fee-for-service payment mechanism, it pays the same facility 10,000 KES per delivery for members in the national scheme using a case-based payment system. These multiple provider payment mechanisms and payment rates may generate conflicting and unwanted incentives for providers.^20^ There is evidence that these incoherent provider payment mechanisms have resulted in preferential treatment of civil servants at the expense of non–civil servants.^19,20^ This includes practices like sending non–civil servant NHIF members to purchase medicines from private pharmacies outside the hospital using out-of-pocket payments, while providing medicines to civil servants within the hospital because of the perception that the capitation rate for non–civil servants was inadequate.^19^ Multiple payment mechanisms therefore potentially incentivize unfairness in the system.

#### Efficiency

**Figure 4:**
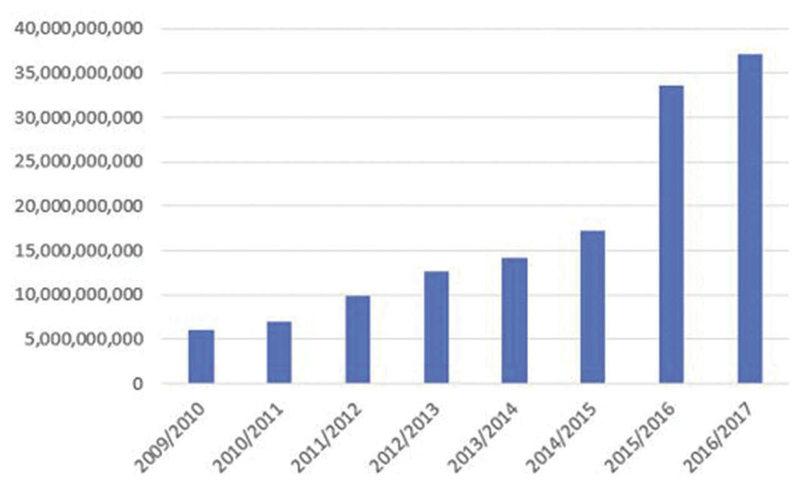
NHIF Revenue Collection in Absolute Terms by Year

There are a number of efficiency concerns for the NHIF. First, eventhough the annual revenue collection by the NHIF increased sixfold from 5.9 billion KES in fiscal year 2009–2010 to 37 billion KES in fiscal year 2016–2017 (Figure 4),^23,25,27,28^ this amount represented approximately 5% of the country’s current health expenditure.^51^ This implies that the NHIF is not an efficient mobilizer of revenues for health care because of the feasibility challenges discussed previously.

**Figure 5:**
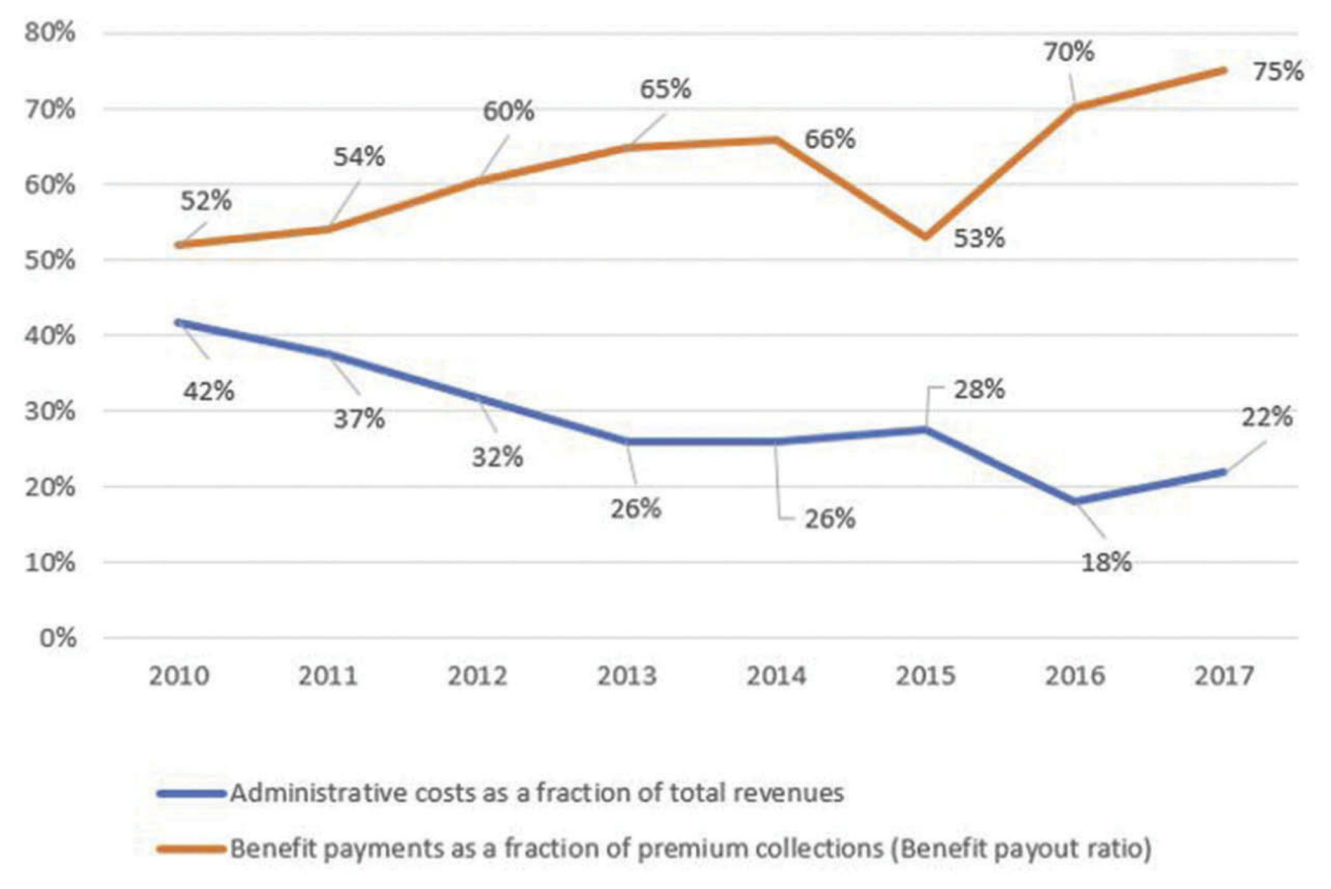
NHIF Administrative Cost and Benefit Payout Ratio

Second, though administrative costs as a share of total revenues were reduced from 42% to 22% and the benefit payout ratio increased from 52% to 75% between 2010 in 2017 (Figure 5),^23,25,27,28^ these indicators are still poor, indicating persistent operational inefficiencies. An analysis of NHIF reports reveals that staff costs contributed 63% of administative costs in fiscal year 2016–2017, indicating that staffing is a key driver of operational inefficiencies of the NHIF. This is consistent with the findings of the strategic review.

Third, NHIF’s fragmented risk pools also contribute to the inefficiency of the NHIF. The NHIF operates three schemes (CSS, the national scheme, and HISP), each offering different benefit packages.^19,28,35^ Though each of these packages includes inpatient and outpatient care, there is considerable variation between them.^19^ The multiple benefit packages and fragmentation of risk pools undermine risk-sharing and income cross-subsidization,^4,52^ resulting in higher risk-adjusted costs coverage than would have existed under a larger pool, thus compromising technical efficiency.^4^

Fourth, the voluntary nature of informal sector individual membership has left the NHIF susceptible to adverse selection. For instance, it has been reported that health care providers encourage and even facilitate the enrollment of patients in need of long-term inpatient care or expensive procedures.^20^

Fifth, weak accountability mechanisms have led to an increase in cases of fraud by the NHIF and health care providers.^19^ Fraud leads to leakage of resources, which results in inefficiencies.

Sixth, it has been reported that the NHIF has poor quality assurance mechanisms that have resulted in purchasing of poor quality of care.^7^ For instance, though in theory the NHIF is meant to carry out regular monitoring of health care facilities and conduct clinical audits to check on quality of care, in practice these activities are infrequent.^7^ Spending scarce resources on poor-quality care compromises techncial efficiency. Though an adequacy assessment of the SafeCare program reported that the median quality score of enlisted facilities improved from 41% (interquartile range 33%–51%) to 51% (interquartile range 44%–63%),^17^ the number of health care facilities that were enlisted in the SafeCare program was low (852 health care facilities as of 2017).^17^

Finally, allocating resources preferentially to hospitals that are predominantly located in urban areas rather than the more cost-effective primary health care services compromises allo-cative efficiency.

#### Sustainability

The expanded benefit package offered by the NHIF coupled with the upward revision of provider reimbursement rates is unsustainable. The upward revision of inpatient reimbursement rates was a result of lobbying by private health care providers as a condition for accepting the introduced capitation rates.^39^ Private health care providers are a powerful interest group in the Kenyan health policy landscape and are represented in the NHIF management board by professional associations. Their influence on the NHIF with regard to reimbursement rates represents what we will call here “purchaser capture,” a situation in which the actions of a purchaser are influenced by, and in favor of, health care providers. Table 5 presents a hypothetical scenario in which the NHIF recruits one million more members from the informal sector population, a more than 100% increase from the current rate.

Scenario one is an optimistic scenario. Under this scenario, at a monthly premium rate of 500 KES, the annual revenues will be six billion KES. Assuming a very conservative dependency ratio of two beneficiaries for each principal member (the average household size in Kenya is four), the total number of new NHIF members entitled to benefits will be three million. At the current annual capitation rate of 1,200 KES, the NHIF will be required to pay 3.6 billion KES to facilities annually for its newly registered members.

According to the last financial report, the NHIF paid an average of 1,475 KES annually for inpatient claims per registered member.^28^ At this rate, the NHIF will need to pay 4.42 billion KES for inpatient claims. The total annual expenditure (payments to health care providers plus administrative cost) will therefore be 8.5 billion KES against total annual revenues of six billion KES, which gives a deficit of two billion KES (25%). A modest administrative charge of 7.5% increases this deficit to 2.5 billion KES (29%). Under scenario two in which the current NHIF level of administative cost (22%) and the current assumption used by NHIF for dependency ratio (four dependents per principal member), the deficit increases to 59%.

Although in the current membership mix, where the majority of NHIF members are formal sector employees, the deficit from the informal sector risk segment will likely be offset by the revenues from the formal sector segment, the calculus will tip in the direction of financial deficits and unsustainability when more informal sector members are registered, as formal sector worker enrollment remains constant. Thus, the assumption that the formal sector contributions cushion the NHIF from financial collapse is probably overly optimistic. Though we do not have access to data on revenues from the formal sector, the structure of incomes in Kenya is such that a majority of Kenyans earn very low salaries, which implies that the average contribution from formal sector workers may be just slightly above 500 KES.

## Discussion

The implementation of these reforms demonstrates both commitment and political will by the Kenyan government to steer the country toward UHC. The reforms implemented by the NHIF resulted in several positive outcomes. However, our analysis shows that these improvements are not sufficient. For instance, though population coverage by the NHIF has increased, it remains considerably low at 14%. This resonates with findings from other LMICs that have attempted to expand health insurance coverage using a voluntary mechanism.^4^ Though the NHIF has doubled its revenue collection, this amounted to only 5% of Kenya’s total health expenditure.^51^ There is overwhelming evidence from other settings that voluntary mechanisms do not mobilize sufficient resources for health.^53,54^ Though the NHIF has reduced its administrative costs by half, at 22% the NHIF is still highly inefficient. This level is much higher compared to other social health insurers globally. For instance, an analysis of administrative costs of insurance schemes in 58 countries found that the average level of administrative costs among public insurance schemes was 4.7%.^32^

Further, when the NHIF reforms are examined, it is clear that there are concerns regarding the feasibility, equity, efficiency, and sustainability of the Kenyan government’s policy decision to move toward NHIF using a voluntary contributory mechanism. These findings mirror the results in other settings that operate contributory mechanisms.^53^ It is important that policy design and implementation are aligned with “best practice” and enhance the country’s aspiration to achieve UHC. Several policy actions are imperative.

First, regarding revenue collection, Kenya will not mobilize sufficient resources using a voluntary contributory mechanism. Though NHIF can feasibly mobilize resources from formal sector workers through payroll deductions, expanding national pools through public subsidy is key to expanding population coverage with prepayment financing in a setting like Kenya that is characterized by high informality and poverty. Kenya should consider allocating tax revenues to the NHIF to provide coverage to Kenyans. To do this, robust actuarial analysis should be conducted to inform the estimates on the resource requirements for the NHIF. One option could be to use tax funds to provide full subsidies for the poor and partial subsidies for the rest of the informal sector with some ability to pay premiums. To provide subsidies for the poor, Kenya will need to develop and implement a framework for targeting/identification of the poor at scale. Without a national framework for poverty identification, it will be impossible to scale up a health insurance subsidy program for the poor.

This option comes with several caveats. First, several targeting approaches vary in inclusion and exclusion errors, which have implications for who benefits from an intervention targeted at the poor.^55,56^ Targeting mechanisms also require robust capacity in technical skills, information systems, and verification. It has also been argued that targeting may not always be cost-effective and may lead to poor-quality service delivery. ^7^ The capacity to implement a targeting mechanism and the cost-effectiveness of a targeting approach must hence be assessed to inform a decision to target subsidies.

An alternative approach for the use of tax funds is to adopt a universal approach and provide subsidies to everyone who is uninsured (informal sector and the poor). Such an approach should, however, be weighed against the fiscal capacity to do so. Unlike contributory health insurance, a tax funding approach has the potential to expand coverage faster and may be more administratively efficient. This approach will not only resolve the challenge of expanding population coverage but also resolve the challenge of the financial unsustainability of the NHIF. Rather than playing both revenue collection (by collecting premiums from individuals) and purchasing roles, the NHIF’s mandate could be restricted to strategic purchasing, with revenues collected through direct and indirect taxes by the country’s tax collecting agency and allocated to the NHIF to purchase services for Kenyans.

Secondly, with regard to risk pooling, the NHIF should consider consolidating the CSS, national scheme, and HISP scheme into one pool. This will allow for greater cross-subsidization and minimize administrative costs.

Third, with regard to purchasing, and related to risk pooling, consolidation of the risk pools has to be accompanied by harmonization of benefit packages. We recognize that this might be politically difficult, especially if it means reducing benefits that certain groups, such as civil servants, are entitled to. However, schemes with comparable benefit packages could be harmonized initially and a policy decision made not to introduce new benefit packages but rather to progressively review and update the existing ones toward harmonization.

Fourth, NHIF should adopt similar provider payment rates for similar services to minimize the generation of perverse incentives.

Fifth, the determination of provider payment rates should be informed by evidence generated from rigorous costing and actuarial analysis, rather than recommendations from health care providers. The NHIF should avoid what we call here purchaser capture, where health care providers exert a high influence on provider payment rates, resulting in inflated costs of services, that benefit providers but compromise the sustainability of the NHIF. Appropriately costed provider payment rates will enhance the financial sustainability of the NHIF.

Sixth, significant capacity is required to strengthen the delivery of services to its members. Specifically, the NHIF will require expanding the network of health care facilities contracted to provide services to its members. In doing this, attention should be paid to contracting facilities in poor, rural, and/or marginalized areas to remedy the pro-urban and pro-rich geographical distribution of contracted facilities.

Finally, the NHIF can strengthen its quality management processes and the enforcement of contract terms such that providers are held accountable for providing good quality services to its members.

## Figures and Tables

**Table 1:** Selected Health Financing Indicators for Kenya^3^

Health financing indicators	2002–2003	2005–2006	2008–2009	2013–2014	2015–2016
Percentage of population with health insurance coverage	9.7	n/a	10.0	17.1	19
Percentage of total health expenditure financed by public sources	29.6	29.3	28.8	33.5	37
Percentage of total health expenditure financed by donors	16.4	31.0	34.5	24.7	23.4
Percentage of total health expenditure financed by private sources	54.0	39.3	36.7	40.6	39.6
Percentage of total health expenditure paid for through out-of-pocket expenditure	n/a	n/a	25.1	26.6	26.1
Total health expenditure per capita (USD)	51.2	59.5	66.3	77.4	78.6
Government health expenditure as % of total government expenditure	7.9	5.1	4.8	6.1	6.7
Total health expenditure as % of gross domestic product	5.1	4.7	5.4	6.8	5.2
Public expenditure on health as % of gross domestic product	1.5	1.4	1.6	2.3	2.2

**Table 2:** Documents and Papers Included in the Document Review

	Author (date)	Study/report title	Study/report objective
Peer-reviewed papers			
1	Abuya et al.^10^	Historical Account of the National Health Insurance Formulation in Kenya: Experiences from the Past Decade	To trace the historical process of the development of the National Health Insurance Scheme proposal and illuminates factors that led to the failure of implementing the policy
2	Barasa et al.^11^	Extending Voluntary Health Insurance to the Informal Sector: Experiences and Expectations of the Informal Sector in Kenya	To examine the experiences and perceptions of informal sector individuals regarding membership with the NHIF
3	Kazungu and Barasa^9^	Examining Levels, Distribution and Correlates of Health Insurance Coverage in Kenya	To examine the levels, inequalities, and factors associated with health insurance coverage in Kenya
4	Munge et al.^7^	A Critical Analysis of Purchasing Arrangements in Kenya: The Case of the National Hospital Insurance Fund	To critically analyse purchasing arrangements in Kenya, using the NHIF as a case study
5	Oketch and Lelengwe^12^	Analysis of Universal Health Coverage and Equity on Health Care in Kenya	To critically review the various initiatives that the government of Kenya has initiated over the years toward the realization of UHC and how this has impacted health equity
6	Okungu et al.^13^	Extending Coverage to Informal Sector Populations in Kenya: Design Preferences and Implications for Financing Policy	To document the views of informal sector workers regarding different prepayment mechanisms and critically analyze key design features of a future health system and the policy implications of financing UHC in Kenya
7	Sieverding et al.^14^	Private Healthcare Provider Experiences with Social Health Insurance Schemes: Findings from a Qualitative Study in Ghana and Kenya	To explore private providers’ perceptions of and experiences with participation in two different social health insurance schemes in sub-Saharan Africa—the National Health Insurance Scheme in Ghana and the NHIF in Kenya
Grey literature			
8	Gesellschaft für Internationale Zusammenarbeit (GIZ)^15^	*Willingness and Ability to Pay for the NHIF Premium among the Informal Sector*	To examine the willingness and ability to pay the NHIF premium among the informal sector in Kenya
9	IFC^8^	*Strategic Review of the National Hospital Insurance Fund*	To carry out a comprehensive strategic review of NHIF and a market assessment of prepaid health schemes/ health maintenance organisations in Kenya
10	IFC^16^	*HISP Process Evaluation Report*	To evaluate the implementation process of the HISP pilot
11	IFC^17^	*NHIF–SafeCare Program End Term Evaluation Report*	To evaluate the NHIF–SafeCare program
12	Kimani et al.^18^	*Baseline Survey of the Health Insurance Subsidy Programme*	To examine the baseline characteristics of HISP beneficiaries in Kenya
13	Mbau et al.^19^	*Strategic Purchasing in Healthcare in Kenya: Examining Purchasing Reforms by the National Hospital Insurance Fund*	To examine how recent NHIF reforms have influenced the ability of the NHIF to purchase health care services strategically
14	Mbau et al.^20^	*Examining Multiple Funding Flows to Healthcare Facilities in Kenya*	To examine how multiple funding flows to health care facilities have influenced provider behavior in Kenya
15	NHIF^21^	*Civil Servants Scheme Operations Manual*	To outline the operational arrangement and implementation plan of the civil servants program
16	NHIF^22^	*Health Insurance Subsidy for the Poor (HISP) Operations Manual*	To outline the operational arrangement and implementation plan of the HISP program
17	NHIF^23^	*NHIF Management Report (2013–2014)*	To analyze and present the performance of the NHIF for fiscal year 2013–2014
18	NHIF^24^	*NHIF Strategic Plan 2014–2018*	To outline strategic objectives of the NHIF over the period 2014–2018
19	NHIF^25^	*NHIF Management Report (2014–2015)*	To analyze and present the performance of the NHIF for fiscal year 2014–2015
20	NHIF^26^	*NHIF Informal Sector Strategy*	To outline strategic objectives of the NHIF during the period 2016–2018 with regard to expanding membership among the informal sector
21	NHIF ^27^	*NHIF Management Report (2015–2016)*	To analyze and present the performance of the NHIF for fiscal year 2015–2016
22	NHIF^28^	*NHIF Management Report (2016–2017)*	To analyze and present the performance of the NHIF for fiscal year 2016–2017
23	The World Bank^29^	*Impact Evaluation of the Health Insurance Subsidy Program in Kenya*	To examine the effectiveness of the health insurance subsidy program in increasing health care utilization and financial risk protection among the poor in Kenya

**Table 3:** Revisions of NHIF Contribution Rate

Old income groups and premium contribution rates (KES)		New income groups and premium contribution rates (KES)		
Monthly salary	Monthly premium	Income group	Premium	% increase
1,000–1,499	30	Less than 5,999	150	400
1,500–1,999	40			275
2,000–2,999	60			150
3,000–3,999	80			88
4,000–4,999	100			50
5,000–5,999	120			25
6,000–6,999	140	6,000–7,999	300	114
7,000–7,999	160			88
8,000–8,999	180	8,000–11,999	400	122
9,000–9,999	200			100
10,000–10,999	220			82
11,000–11,999	240			67
12,000–12,999	260	12,000–14,999	500	92
13,000–13,999	280			79
14,000–14,999	300			67
15,000 and above	320	15,000–19,999	600	88
		20,000–24,999	750	134
		25,000–29,999	850	166
		30,000–34,999	900	181
		35,000–39,999	950	197
		40,000–44,999	1,000	213
		45,000–49,999	1,100	243
		50,000–59,999	1,200	275
		60,000–69,999	1,300	306
		70,000–79,999	1,400	338
		80,000–89,999	1,500	369
		90,000–99,999	1,600	400
		Over 100,000	1,700	431
Informal sector	160		500	213

**Table 4:** NHIF Reimbursement Rates^28^

Provider payment method	Benefit covered	Reimbursement rate
Capitation	Outpatient services for national scheme, sponsored scheme and civil servants of job groups A–K (outpatient services include consultation, treatment, basic diagnostic tests: laboratory and X-ray; day care surgery and drugs under the Kenya Essential Drug List of 2010)	1,400 KES per beneficiary per year
Case-based payment	Maternity package (national and sponsored schemes)	Normal delivery 10,000 KES
		Caesarean section 30,000 KES
	Free maternity program	Normal delivery and caesarean section 5,000 KES
	Renal dialysis	9,500 KES per session twice weekly
		Includes pre-dialysis, intra-dialysis session, and post-dialysis care
	Surgical package	Major surgeries: 80,000 KES (levels three and four) 130,000 KES (levels five and six)
		Minor surgeries: 30,000 KES (levels three and four) 40,000 KES (levels five and six)
Fee-for-Service	Radiology package	Magnetic resonance imaging capped at 15,000 KES
		Computed tomography scan capped at 8,000 KES
	Dental	Capped at 40,000 KES
	Optical	Capped at 50,000 KES
	Maternity for managed schemes	Capped at 200,000 KES
	Outpatient services for civil servants of job groups L and above	Job group L capped at 100,000 KES
		Job group M capped at 150,000 KES
		Job group N capped at 200,000 KES
		Job group P capped at 225,000 KES
		Job group Q capped at 250,000 KES
		Job groups R, S, T capped at 350,000 KES
	Inpatient services for civil servants of job groups L and above	Job group L capped at 1,000,000 KES
		Job group M capped at 1,250,000 KES
		Job group N capped at 1,500,000 KES
		Job group P capped at 1,750,000 KES
		Job group Q capped at 2,000,000 KES
		Job groups R, S, T capped at 2,250,000 KES
Rebate (per diem)	Covers admitted medical and surgical conditions	2,000–4,000 KES per day (no copayments in public facilities)

A job group represents seniority and corresponding salary scales where a higher alphabet represents more seniority

**Table 5:** NHIF Annual Cash Flow Outlook for a Hypothetical Population of One Million Informal Sector Principal Members (in KES)^a^

	Scenario 1	Scenario 2
Number of principal members	1,000,000	1,000,000
Dependency ratio	2	4
Number of dependents	2,000,000	4,000,000
Total membership	3,000,000	5,000,000
Monthly premium contribution	500 KES	500 KES
Annual premium contribution	6,000 KES	6,000 KES
Total annual premium contribution	6,000,000,000 KES	6,000,000,000 KES
Annual outpatient capitation rate	1,200 KES	1,200 KES
Total annual capitation paid	3,600,000,000 KES	6,000,000 KES
Annual inpatient claim	1,475 KES	1,475 KES
Total annual inpatient claim	4,425,000,000 KES	7,375,000,000 KES
Percentage of administrative cost	7.5%	22%
Total annual administrative cost	450,000,000 KES	1,320,000,000 KES
Total payout	8,475,000,000 KES	14,695,000,000 KES
Net annual cash flows	2,475,000,000 KES	8,695,000,000 KES
% Deficit	29%	59%

a Assumes current level of administative costs and the NHIF assumption for dependency ratio.
